# Exchanging dietary fat source with extra virgin olive oil does not prevent progression of diet-induced non-alcoholic fatty liver disease and insulin resistance

**DOI:** 10.1371/journal.pone.0237946

**Published:** 2020-09-03

**Authors:** Dragana Rajcic, Annette Brandt, Cheng Jun Jin, Victor Sánchez, Anna Janina Engstler, Finn Jung, Anika Nier, Anja Baumann, Ina Bergheim

**Affiliations:** 1 Department of Nutritional Sciences, Molecular Nutritional Science, University of Vienna, Vienna, Austria; 2 Institute of Nutrition, SD Model Systems of Molecular Nutrition, Friedrich-Schiller University of Jena, Jena, Germany; Medizinische Fakultat der RWTH Aachen, GERMANY

## Abstract

Dietary fat is discussed to be critical in the development of non-alcoholic fatty liver disease. Here, we assess the effect of exchanging dietary fat source from butterfat to extra virgin olive oil on the progression of an already existing diet-induced non-alcoholic fatty liver disease in mice. Female C57BL/6J mice were fed a liquid butterfat-, fructose- and cholesterol-rich diet (BFC, 25E% from butterfat) or control diet (C, 12%E from soybean oil) for 13 weeks. In week 9, fat sources of some BFC- and C-fed mice were switched either to 25E% or 12E% olive oil (OFC and CO). Glucose and insulin tolerance tests were performed, and markers of liver damage and glucose metabolism were assessed. After 6 weeks of feeding, BFC-fed mice had developed marked signs of insulin resistance, which progressed to week 12 being not affected by the exchange of fat sources. Liver damage was similar between BFC- and OFC-fed mice. Markers of lipid metabolism and lipid peroxidation in liver and of insulin signaling in liver and muscle were also similarly altered in BFC- and OFC-fed mice. Taken together, our data suggest that exchanging butterfat with extra virgin olive oil has no effect on the progression of non-alcoholic fatty liver disease and glucose tolerance in mice.

## Introduction

Insulin resistance is acknowledged as one of the key risk factors in the development of several metabolic diseases, among them also non-alcoholic fatty liver disease (NAFLD) [1, 2]. Results of epidemiological studies suggest that NAFLD by now affects ~25% of the general global population and, depending on the area of the world studied, ~55–68% of patients with type 2 diabetes, making NAFLD the most prevalent liver disease in the world [3, 4]. Indeed, studies also suggest that NAFLD soon will be the leading cause of liver transplantation in the US [5] and annual direct medical costs associated with the disease are estimated to account to approximately $1,613 per patient in the US and €354 to €1,163 per patient in Europe [6]. Despite being intensely studied, molecular mechanisms involved are still not fully understood and universally accepted therapies other than life-style interventions are still not available. However, the latter are often associated with low compliance and high relapse rates [7]. Also, with respect to dietary interventions and recommendations focusing on changes of nutritional intake, the role of different foods and nutrients like sugar or fat in disease development as well as therapy is still a matter of debate.

Besides genetic predisposition, overnutrition along with overweight and obesity have been identified as major factors contributing to the development of NAFLD (for overview see [8]). In more recent years, not only overnutrition per se but rather dietary patterns associated with the increased caloric intake are discussed to be critical in the development of the disease [9, 10]. Indeed, a so-called `Western-type´ dietary pattern rich in processed foods, sweets, sugar-sweetened beverages, red meat and refined grains resulting in a high intake of mono- and disaccharides as well as saturated fats and a low fiber intake are thought to be widely contributing to the onset and progression of NAFLD [11–13]. Results of meta-analyses further suggest that altering dietary fat intake through changes in fat sources or through supplementing n3 fatty acids resulting in an increase in the ratio of n3 to n6 fatty acids in diet may improve liver parameters of NAFLD patients [14, 15]. A Mediterranean diet being rich in fruits, vegetables, nuts, beans and whole grains but also using olive oil as predominate source of oil has also been suggested to be beneficial in the treatment of NAFLD patients [16–18]. As part of a Mediterranean diet, olive oil has been associated with benefits on human health, and herein, especially metabolic diseases and NAFLD [19, 20]. Also, olive oil and compounds found in olive oil e.g. fatty acids and polyphenols are discussed to exert beneficial effects in settings of insulin resistance and NAFLD (for overview see [17, 21]); however, to date, data from animal and human studies evaluating the isolated effects of olive oil on the liver and/ or insulin resistance are limited and at times contradictory. Indeed, while some studies suggest that polyphenol-rich extra virgin olive oils may possess beneficial effects in regard to the development of liver diseases, and herein, especially NAFLD [22], results of other studies reported no or even adverse effects [23, 24].

Starting from this background, the aim of the present study was to assess if exchanging butterfat in a fat-, fructose- and cholesterol-rich diet with extra virgin olive oil in mice with a diet-induced NAFLD and insulin resistance has beneficial effects on markers of diet-induced NAFLD and insulin resistance. Accordingly, to induce NAFLD, mice were pair-fed a liquid diet rich in butterfat, fructose, and cholesterol for 8 weeks. It has been shown before by us that when feeding this kind of diet even `normo-caloric´, mice develop macrovesicular fat accumulation and early signs of inflammation in the liver within 6–8 weeks which progress to beginning of non-alcoholic steatohepatitis (NASH) within additional 5 weeks of feeding this diet [25]. To determine the effects of extra virgin olive oil on the progression of NAFLD and insulin resistance, in some mice, dietary butterfat was completely exchanged with the oil in week 9 for the next 5 weeks while the composition of the diet was maintained otherwise. Markers of insulin resistance and NAFLD were assessed and compared to those determined in mice fed a butterfat-rich diet during the entire time.

## Materials and methods

### Animals and feeding

Female C57BL/6J mice obtained from Janvier (SAS, Le Genest-Saint-Isle, France) were used in the mouse feeding experiments. In previous studies of our group but also other groups it was shown that female mice are more or similarly susceptible to the development of early stages of fructose-induced NAFLD [26, 27]. Also, results of others suggest a close resemblance of variability of traits and parameters between male and female mice [28]. All procedures were approved by the local institutional animal care and use committee (‘Landesamt für Verbraucherschutz’, reference number: 02-004/16, Thuringia, Germany) and animals were handled in accordance to the European Convention for the Protection of Vertebrate Animals used for Experimental and Other Scientific Purposes. Mice were housed under controlled conditions in a pathogen-free barrier facility at the Friedrich-Schiller University of Jena, Germany, accredited by the Association for Assessment and Accreditation of Laboratory Animal Care and had free access to water at all times. Experimental setup used in the present study and sample size calculations were based on previous studies of our group [25]. Indeed, in these studies, it was shown that in this feeding model, 5 weeks of treatment with a compound/ oil are sufficient to show `therapeutic´ efficacy [25, 29]. For the first 8 weeks of the experiment, animals were either fed a liquid control diet (C; 69E% carbohydrates, 12E% fat derived from soybean oil, 19E% protein; Ssniff, Soest, Germany) or a liquid butterfat-, fructose- and cholesterol-rich diet (BFC; 60E% carbohydrates, 25E% fat derived from butterfat, 15E% protein, 50% wt/wt fructose and 0.16% wt/wt cholesterol; Ssniff, Soest, Germany). Mice were then randomly assigned to the following groups (n = 7-8/ group): mice continuously fed the C or BFC diet or mice switched to a control diet with exchanged fat source (CO, 12E% from extra virgin olive oil instead of soybean oil) or a BFC diet with exchanged fat source (OFC, 25E% from extra virgin olive oil instead of butterfat). Study design is summarized in S1 Fig. Composition of diets used in the feeding trail are summarized in S1 Table and were previously described in detail [30]. Mice fed control diets and those fed the fat-, fructose- and cholesterol-rich diets, respectively, were pair-fed to ensure equal caloric intake also detailed previously [25]. Dietary intake was assessed daily, and body weight was assessed weekly. In weeks 7–8 and 12–13 of feeding, mice were fasted for 6 h, anesthetized intraperitoneally (i.p) with ketamine/xylazine solution and an insulin tolerance test (ITT) or glucose tolerance test (GTT) were performed as described before [25]. After 13 weeks of feeding, mice were again anesthetized with ketamine/xylazine (i.p.), and after a terminal blood collection from portal vein, animals were killed by cervical dislocation, and liver and muscle tissue were collected.

### Assessment of liver damage and inflammation

To assess liver damage, sections of paraffin-embedded livers (4 μm) were stained with hematoxylin and eosin and histology was scored using the NAFLD activity score (NAS) as described by others [31]. Alanine transaminase (ALT) and aspartate transaminase (AST) activity in plasma was measured in a routine laboratory (University Hospital Jena, Jena, Germany).

### Staining of 4-hydroxynonenal protein adducts, neutrophils and F4/80 positive cells

To assess 4-hydroxynonenal protein adducts (4-HNE) and F4/80 positive cells in paraffin-embedded liver sections (4 μm), immunohistochemical stainings (4-HNE: 1:1000 polyclonal rabbit antibody (#H-1110) AG Scientific, San Diego, CA, USA; F4/80: 1:200 monoclonal rat antibody (ab6640) Abcam, Cambridge, United Kingdom) were performed using an peroxidase-linked secondary antibodies and diaminobenzidine as described in detail before [32, 33]. For staining of neutrophil granulocytes in liver sections a commercially available staining kit was used (Naphthol AS-D Chloroacetate kit, Sigma-Aldrich, Steinheim, Germany) as previously described [34]. 4-HNE staining was quantified using an analysis system (Leica Application Suite, Leica, Wetzlar, Germany), and numbers of F4/80 positive cells as well as neutrophil granulocytes were counted per microscopic field (400 x or 200 x, respectively) utilizing a microscope integrated camera (Leica DM4000 B LED, Leica, Wetzlar, Germany), as detailed previously [34, 35].

### Free fatty acids in plasma and triglycerides in liver tissue

For determining levels of free fatty acids in plasma, a commercially available kit (Free Fatty Acid Quantitation Kit, Sigma-Aldrich, Steinheim, Germany) was used according to manufacturer’s instructions. Measurement of triglyceride levels in liver tissue was performed as previously described in detail [34]. Measured values were normalized to protein concentration.

### RNA isolation and real-time RT-PCR

RNA was isolated from liver and muscle tissue using peqGOLD Trifast (Peqlab, Erlangen, Germany), concentration of RNA was measured, and cDNA was synthetized (Reverse Transcription System, Promega GmbH, Madison, WI, USA). Using primers listed in S2 Table, real-time polymerase chain reaction (PCR) was performed to evaluate expression of respective genes normalized to 18S expression as previously described in detail [33].

### PPARγ activity assay

Whole-cell extracts were isolated from frozen liver tissue using a commercially available extraction buffer according to the instructions of the manufacturer (Active Motif, Inc., Carlsbad, CA, USA). Due to a lack of suitable tissue samples, this was only possible for n = 3–4 mice per group. Peroxisome proliferator-activated receptor gamma (PPARγ) activity was measured with a commercially available kit in accordance with the protocol provided by the manufacturer (PPARγ Transcription Factor Assay Kits, Active Motif, Inc., Carlsbad, CA, USA).

### Western blot

Cytosolic protein extracts were isolated from liver tissue and separated in SDS-PAGE as detailed before [34]. After transferring proteins to a polyvinylidene difluoride membrane (Bio-Rad Laboratories, Hercules, CA, USA), and incubating them with primary antibodies detecting cleaved caspase 3 (Cell Signaling Technology, Massachusetts, USA) or β-actin (Santa Cruz Biotechnology, Dallas, Texas, United States), bands were detected using Super Signal West Dura kit (Thermo Fisher Scientific, Waltham, MA, USA) [34, 36] and analyzed using the ChemiDoc XRS System with Image Lab software (Bio-Rad Laboratories, Hercules, CA, USA).

### Statistical analysis

Statistical analysis was performed using PRISM (version 7.03, GraphPad Prism Software, San Diego, CA, USA). A Grubb´s outlier test was performed. An unpaired two-tailed Student’s t-test was used for the comparison of C and BFC groups where applicable, whereas a two-factorial analysis of variance (ANOVA) was carried out for the comparison of four groups after 13 weeks to assess significant differences (p<0.05) followed by Tukey´s post hoc test. Using this approach, the diet effect (DE), olive oil effect (OE) and interaction between diet and olive oil (DExOE) were determined. In case of inhomogeneity of variances data were log-transformed. Data are presented as means ± standard error of mean (SEM). The following labeling was used to identify statistically significant differences between groups: ^a^ p<0.05 compared to C-fed mice, ^b^ p<0.05 compared to BFC-fed mice, ^c^ p<0.05 compared to CO-fed mice.

## Results

### Effect of extra virgin olive oil on body weight gain and markers of insulin resistance

Caloric intake, body weight gain and absolute body weight within control diet (C) and high fat, fructose and cholesterol diet fed groups were similar regardless of olive oil supplementation (see Table 1). Absolute body weight gain and body weight were significantly higher in butterfat-, fructose- and cholesterol-rich diet (BFC) group than in both C and control with extra virgin olive oil (CO) fed mice (p<0.05 for both parameters and groups) after 13 weeks of feeding. Similar differences were not found when comparing control groups with extra virgin olive oil-, fructose- and cholesterol-rich diet (OFC) fed mice.

**Table 1:** Effect of olive oil on caloric intake, body weight, and body weight gain in female mice with early NASH.

Parameter	8 weeks^˅^		13 weeks^˄^				Two-way ANOVA		
	C	BFC	C	BFC	CO	OFC	DEx OE	OE	DE
**Caloric intake (kcal/g bw/d)**	0.43 ± 0.01	0.46 ± 0.01^a^	0.39 ± 0.01	0.44 ± 0.01^a^^,^^c^	0.39 ± 0.01	0.44 ± 0.01^a^^,^^c^	NS	NS	<0.05
**Absolute body weight gain (g)**	3.7 ± 0.2	4.3 ± 0.2	3.6 ± 0.3	5.8 ± 0.4^a^^,^^c^	3.9 ± 0.2	5.0 ± 0.5	NS	NS	<0.05
**Absolute body weight (g)**	21.8 ± 0.3	22.4 ± 0.3	21.6 ± 0.5	23.9 ± 0.6^a^^,^^c^	21.8 ± 0.3	23.0 ± 0.5	NS	NS	<0.05

C, control diet; BFC, butterfat-, fructose- and cholesterol-rich diet; CO, control diet with extra virgin olive oil; OFC, extra virgin olive oil-, fructose- and cholesterol-rich diet; DE, diet effect; OE, olive oil effect; DExOE, interaction between diet and olive oil; NASH, non-alcoholic steatohepatitis; NS, not significant.

^a^ p<0.05 compared to C-fed mice.

^c^ p<0.05 compared to CO-fed mice.

^**˄**^Values are means ± SEM, n = 7–8.

^**˅**^ Unpaired two-tailed Student’s t-test was used to compare groups after 8 weeks.

**Fig 1:**
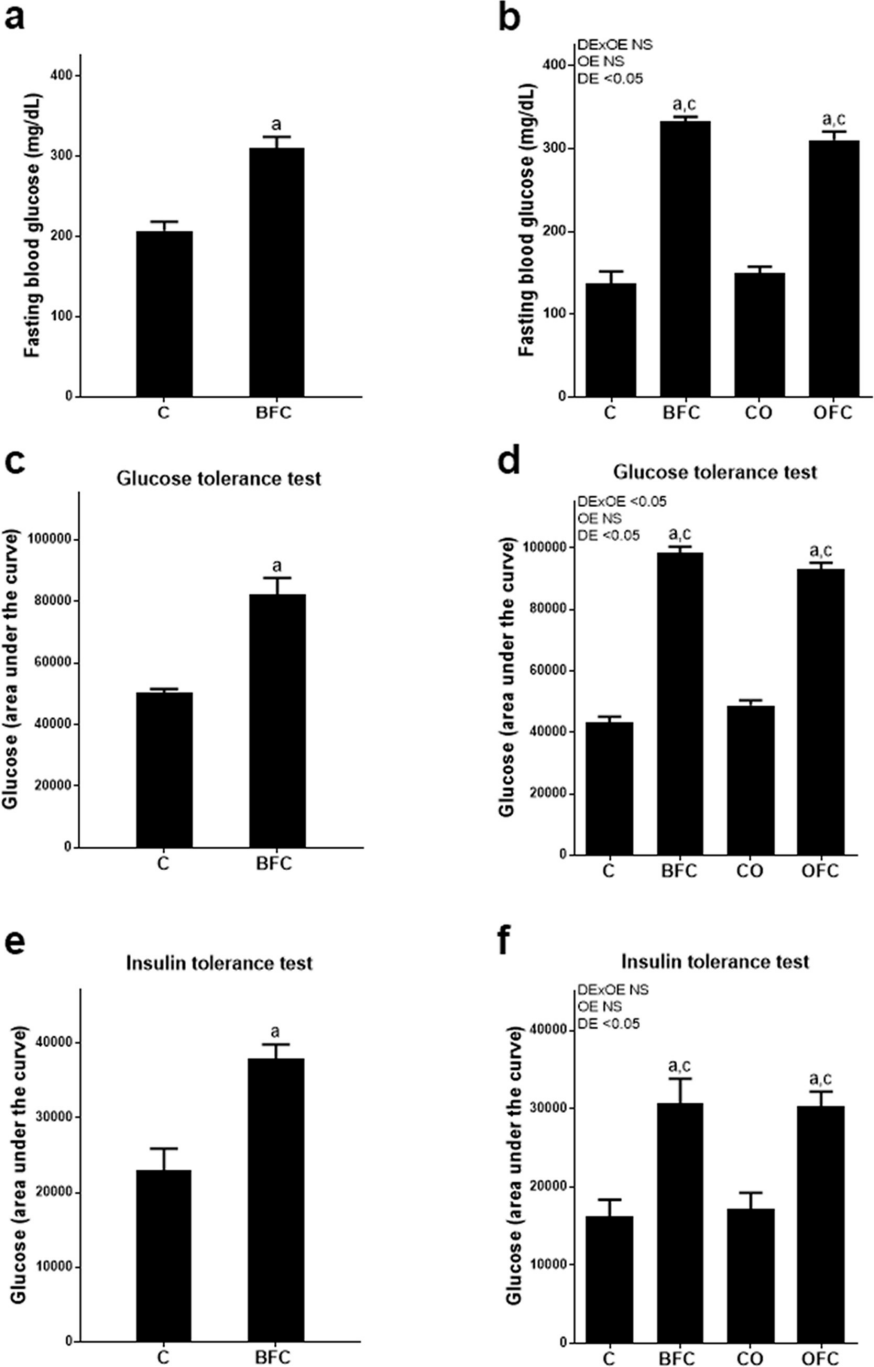
Effect of olive oil on parameters of glucose metabolism in female mice with early NASH. Fasting blood glucose concentration after (a) 7 weeks and (b) 12 weeks of feeding. Area under the curve of glucose levels during the glucose tolerance test after (c) 7 weeks and (d) 12 weeks of feeding, and during the insulin tolerance test of mice after (e) 6 weeks and (f) 11 weeks of feeding. Values are means ± SEM, n = 7–8. Unpaired two-tailed Student´s t-test (p<0.05) was used to compare groups after 6–7 weeks, while two-way ANOVA was used to compare groups after 11–12 weeks of feeding. ^a^ p<0.05 compared to C-fed mice, ^c^ p<0.05 compared to CO-fed mice. C, control diet; BFC, butterfat-, fructose- and cholesterol-rich diet; CO, control diet with extra virgin olive oil; OFC, extra virgin olive oil-, fructose- and cholesterol-rich diet; DE, diet effect; OE, olive oil effect; DExOE, interaction between diet and olive oil; NASH, non-alcoholic steatohepatitis; NS, not significant.

As expected, after 6 and 7 weeks of feeding, respectively, and even more so after 11 and 12 weeks of BFC feeding, fasting glucose levels and area under the curve (AUC) of GTT and ITT were significantly higher in BFC- than in C-fed mice (p<0.05 for both time points and all parameters, see Fig 1). Exchanging butterfat with extra virgin olive oil for the last 5 weeks of the feeding experiment had no effect on the elevation of fasting glucose or AUC of GTT and ITT. Indeed, all these parameters were significantly higher in BFC- and OFC-fed mice than in both control groups while being similar between BFC- and OFC-fed mice. Despite the clear signs of impaired glucose tolerance found in both, the GTT and the ITT, expression of insulin receptor (*Ir*) and insulin receptor substrate 2 (*Irs2*) mRNA was similar between both groups in muscle and liver tissue (see Table 2).

**Table 2:** Effect of olive oil on parameters of glucose metabolism in liver and muscle of female mice with early NASH.

Parameter	13 weeks^˄^				Two-way ANOVA		
	C	BFC	CO	OFC	DEx OE	OE	DE
**Liver**							
***Ir* mRNA (% of control)**	100.0 ± 4.4	76.0 ± 6.3	69.0 ± 12.1	85.8 ± 17.3	NS	NS	NS
***Irs2* mRNA (% of control)**	100.0 ± 15.4	66.6 ± 3.7	80.1 ± 10.2	94.0 ± 24.4	NS	NS	NS
**Muscle**							
***Ir* mRNA (% of control)**	100.0 ± 11.6	66.7 ± 12.9	100.3 ± 17.4	95.6 ± 13.3	NS	NS	NS
***Irs2* mRNA (% of control)**	100.0 ± 36.5	55.5 ± 15.8	61.4 ± 13.4	55.3 ± 7.1	NS	NS	NS

C, control diet; BFC, butterfat-, fructose- and cholesterol-rich diet; CO, control diet with extra virgin olive oil; OFC, extra virgin olive oil-, fructose- and cholesterol-rich diet; DE, diet effect; OE, olive oil effect; DExOE, interaction between diet and olive oil; Ir, insulin receptor; Irs2, insulin receptor substrate 2; NASH, non-alcoholic steatohepatitis; NS, not significant.

^**˄**^Values are means ± SEM, n = 7–8.

### Effect of extra virgin olive oil on markers of liver damage

**Fig 2:**
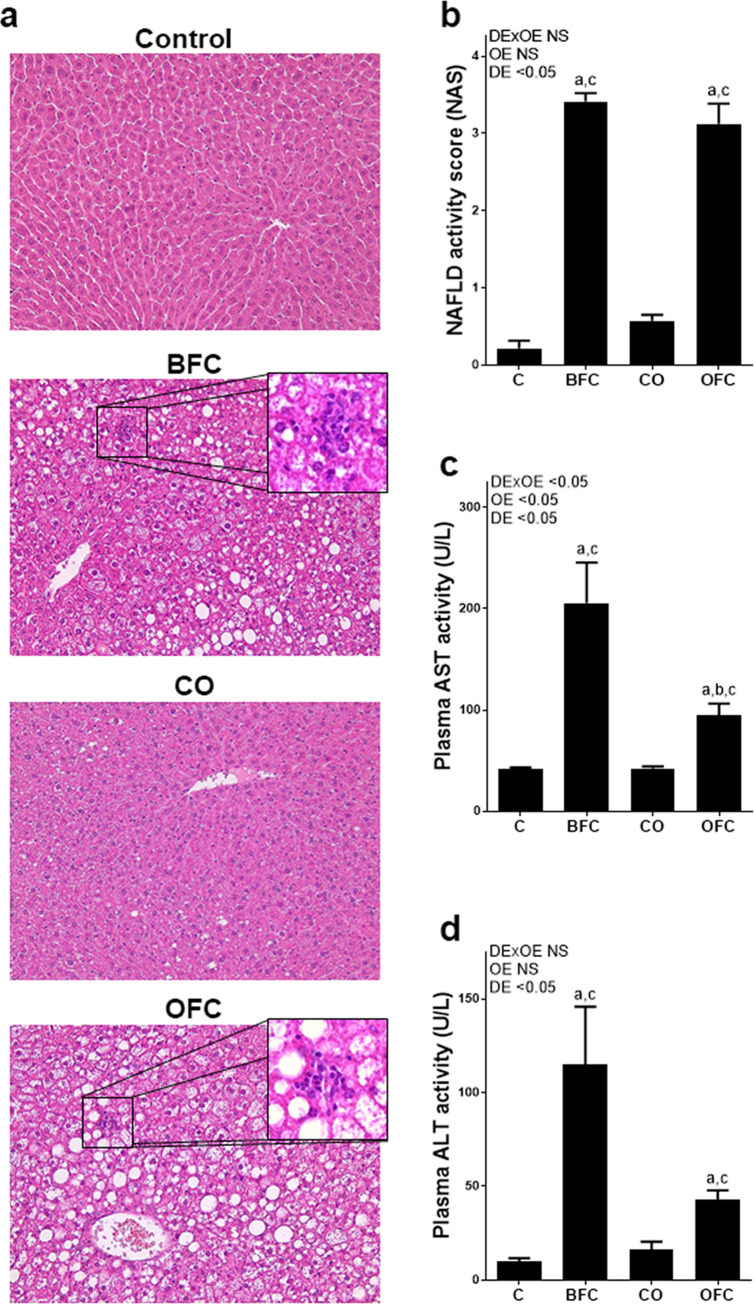
Effect of olive oil on indices of liver damage in female mice with early NASH. (a) Representative photomicrographs of hematoxylin and eosin stained liver sections (200 x, 400 x), and (b) evaluation of liver histology using NAFLD activity score (NAS) adapted from Kleiner et al. [31]. Activity of (c) aspartate aminotransferase (AST) and (d) alanine aminotransferase (ALT) in portal plasma. Values are means ± SEM, n = 6–8. C group for ALT: n = 4 as values were below the detection level. Two-way ANOVA was used to compare groups after 13 weeks of feeding. ^a^ p<0.05 compared to C-fed mice, ^b^ p<0.05 compared to BFC-fed mice, ^c^ p<0.05 compared to CO-fed mice. C, control diet; BFC, butterfat-, fructose- and cholesterol-rich diet; CO, control diet with extra virgin olive oil; OFC, extra virgin olive oil-, fructose- and cholesterol-rich diet; DE, diet effect; OE, olive oil effect; DExOE, interaction between diet and olive oil; NASH, non-alcoholic steatohepatitis; NS, not significant.

Previous studies of our group have shown that within 6–8 weeks of feeding a BFC, mice develop severe steatosis with early signs of inflammation, which progresses to early steatohepatitis after 13 weeks of feeding [37, 38]. In line with our earlier studies, in livers of BFC-fed mice macrovesicular steatosis and inflammatory foci were present (see Fig 2 and Table 3) and NAS as well as hepatic triglyceride levels were significantly higher than those in both control groups (p<0.05 in comparison to both, C- and CO-fed animals and parameters, see Fig 2 and Table 3). Similar to the findings for GTT and ITT, exchanging the fat source to extra virgin olive oil had no effect on liver histology as determined by NAS. Indeed, neither the degree of steatosis nor inflammation as determined by NAS, nor hepatic triglyceride content, differed between BFC- and OFC-fed animals (see Table 3). Liver weight and liver to body weight ratio were also significantly higher in BFC- and OFC-fed animals when compared to both control groups (p< 0.05 for both parameters and groups, see Table 3).

**Table 3:** Effect of olive oil on parameters of liver damage in female mice with early NASH.

Parameter	13 weeks^˄^				Two-way ANOVA		
	C	BFC	CO	OFC	DEx OE	OE	DE
**Liver weight (g)**	0.9 ± 0.03	1.7 ± 0.13^a^^,^^c^	1.0 ± 0.04	1.6 ± 0.05^a^^,^^c^	NS	NS	<0.05
**Liver/body weight ratio (%)**	4.1 ± 0.1	7.1 ± 0.4^a^^,^^c^	4.5 ± 0.1	6.7 ± 0.1^a^^,^^c^	<0.05	NS	<0.05
**NAS Steatosis**	0.2 ± 0.1	3.0 ± 0.1^a^^,^^c^	0.5 ± 0.1	2.5 ± 0.2^a^^,^^c^	NS	NS	<0.05
**NAS Inflammation**	0.0 ± 0.0	0.5 ± 0.1^a^	0.0 ± 0.0	0.6 ± 0.2^a^^,^^c^	NS	NS	<0.05
**Triglycerides (μg/mg protein)**	21 ± 3	111 ± 9^a^	70 ± 20^a^	111 ± 13^a^	<0.05	NS	<0.05
***Il-6* mRNA (% of control)**	100.0 ± 22.1	234.5 ± 32.9^a^	200.1 ± 12.9	238.1 ± 34.5^a^	NS	NS	<0.05
***Tnfα* mRNA (% of control)**	100.0 ± 18.4	188.4 ± 40.0	98.8 ± 25.9	216.0 ± 44.0	NS	NS	<0.05
***Bad* mRNA (% of control)**	100.0 ± 8.0	69.3 ± 7.5^c^	122.4 ± 13.8	83.6 ± 7.2^c^	NS	NS	<0.05
**Cleaved caspase 3 / β-actin (% of control)**	100.0 ± 28.3	157.8 ± 40.3	56.9 ± 14.7	125.9 ± 47.0	NS	NS	NS

C, control diet; BFC, butterfat-, fructose- and cholesterol-rich diet; CO, control diet with extra virgin olive oil; OFC, extra virgin olive oil-, fructose- and cholesterol-rich diet; DE, diet effect; OE, olive oil effect; DExOE, interaction between diet and olive oil; Bad, Bcl2-associated agonist of cell death, Il-6, interleukin 6; NAS, NAFLD activity score; NASH, non-alcoholic steatohepatitis; NS, not significant; Tnfα, tumor necrosis factor alpha.

^a^ p<0.05 compared to C-fed mice.

^c^ p<0.05 compared to CO-fed mice.

^**˄**^Values are means ± SEM, n = 6–8.

**Fig 3:**
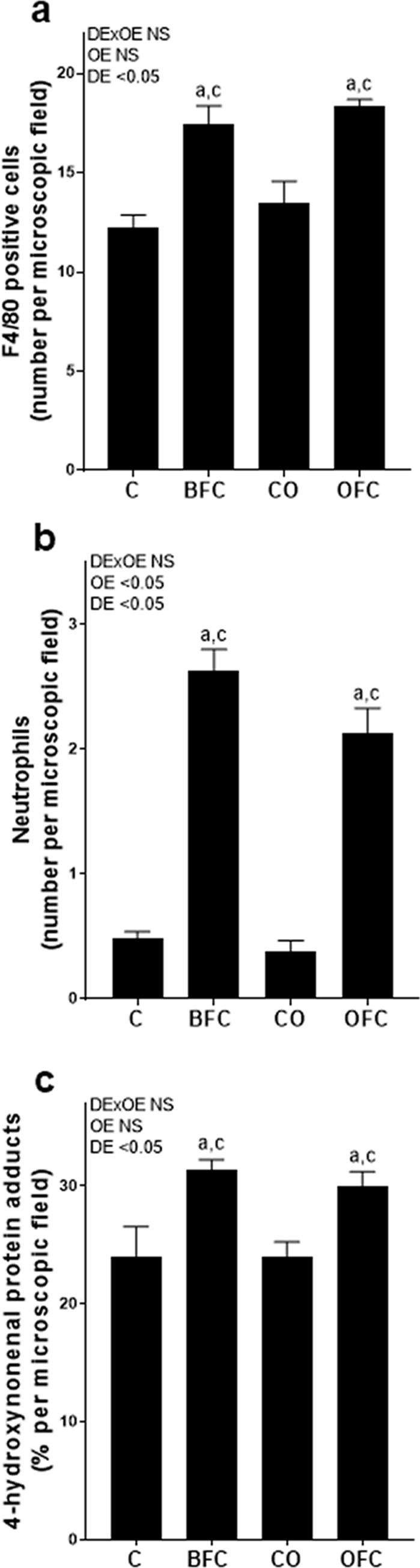
Effect of olive oil on inflammation markers in livers of female mice with early NASH. Number of (a) F4/80 positive cells (400 x), (b) neutrophils (200 x) per microscopic field and (c) densitometric analysis of 4-hydroxynonenal protein adducts in stained liver sections. Values are means ± SEM, n = 7–8. Two-way ANOVA was used to compare groups after 13 weeks of feeding. ^a^ p<0.05 compared to C-fed mice, ^c^ p<0.05 compared to CO-fed mice. C, control diet; BFC, butterfat-, fructose- and cholesterol-rich diet; CO, control diet with extra virgin olive oil; OFC, extra virgin olive oil-, fructose- and cholesterol-rich diet; DE, diet effect; OE, olive oil effect; DExOE, interaction between diet and olive oil; NASH, non-alcoholic steatohepatitis; NS, not significant.

Activities of ALT and AST in plasma were also significantly higher in both BFC- and OFC-fed mice compared to the two control groups; however, AST activity was also significantly higher in plasma of BFC-fed mice when compared to OFC-fed animals (p<0.05, see Fig 2). Numbers of F4/80 positive cells and neutrophils were significantly higher in livers of both BFC- and OFC-fed mice than in both control groups (p<0.05 for both markers and groups compared to C and CO), while these numbers were similar between BFC- and OFC-fed groups (see Fig 3). Concentrations of 4-HNE protein adducts in livers of BFC- and OFC-fed mice were significantly higher than in control groups (p<0.05 for both groups compared to C and CO, see Fig 3). Levels of 4-HNE protein adducts in liver tissue were similar between BFC- and OFC-fed mice. Expressions of interleukin 6 (*Il-6*) and tumor necrosis factor alpha (*Tnfα*) in liver tissue of BFC- and OFC-fed groups were higher than in mice fed control diets; however, as *Tnfα* expression varied considerably, only the difference found for *Il-6* mRNA expression reached the level of significance (C vs. BFC and C vs. OFC both p<0.05, see Table 3). Also, mRNA expression of the pro-apoptotic marker of *Bad* [39] was found to be significantly lower in livers of BFC- and OFC-fed mice, respectively, when compared to CO-fed animals (p<0.05 for both groups compared to CO, see Table 3), while no differences were found between C-fed and BFC- and OFC-fed mice, respectively. Protein levels of cleaved caspase 3 in liver tissue were also similar between groups; however, concentration varied considerably between groups (see Table 3 and S2 Fig).

### Effect of extra virgin olive oil on markers of hepatic lipogenesis

To determine if olive oil intake affected markers of hepatic lipogenesis, mRNA expressions of acetyl- coenzyme A carboxylase (*Acc*), fatty acid synthase (*Fas*), stearoyl- coenzyme A desaturase 1 (*Scd1*), sterol regulatory element-binding protein 1c (*Srebp1c*) and *Pparγ* were assessed in livers of animals. Expressions of *Acc* and *Fas* mRNA were significantly higher in BFC-and OFC-fed mice when compared to the two control groups (p<0.05 for both comparisons of both parameters) but did not differ among each other. Expression of *Scd1* mRNA was significantly higher in livers of BFC- and OFC-fed mice than in C-fed mice (p<0.05 for both comparisons); however, similar differences were not found for the comparison of CO-fed mice and the BFC- and OFC-fed groups. *Srebp1c* mRNA expression and PPARγ activity and mRNA in liver tissue were similar between groups (see Table 4, PPARγ activity; n = 3–4 due to a lack of tissue). Furthermore, plasma levels of free fatty acids were also similar between groups (see Table 4).

**Table 4:** Effect of olive oil on parameters of lipid metabolism in liver and plasma free fatty acid levels of female mice with early NASH.

Parameter	13 weeks^˄^				Two-way ANOVA		
	C	BFC	CO	OFC	DEx OE	OE	DE
***Acc* mRNA (% of control)**	100.0 ± 18.4	445.7 ± 74.6^a^^,^^c^	87.8 ± 14.8	306.4 ± 43.8^a^^,^^c^	NS	NS	<0.05
***Fas* mRNA (% of control)**	100.0 ± 14.6	617.0 ± 64.8^a^^,^^c^	93.6 ± 21.8	482.9 ± 73.2^a^^,^^c^	NS	NS	<0.05
***Scd1* mRNA (% of control)**	100.0 ± 9.8	393.3 ± 66.6^a^	230.6 ± 47.6	279.9 ± 39.1^a^	NS	NS	<0.05
***Srebp1c* mRNA (% of control)**	100.0 ± 9.5	129.8 ± 16.9	101.4 ± 8.4	156.6 ± 26.8	NS	NS	<0.05
***Pparγ* mRNA (% of control)**	100.0 ± 21.4	111.5 ± 11.1	130.0 ± 29.1	103.4 ± 9.3	NS	NS	NS
**PPARγ activity (% of control)**	100.0 ± 32.8	117.5 ± 24.9	98.2 ± 6.3	90.9 ± 7.2	NS	NS	NS
**Free fatty acids in plasma (nmol/μl)**	0.5 ± 0.1	0.3 ± 0.03	0.5 ± 0.1	0.4 ±0.1	NS	NS	NS

C, control diet; BFC, butterfat-, fructose- and cholesterol-rich diet; CO, control diet with extra virgin olive oil; OFC, extra virgin olive oil-, fructose- and cholesterol-rich diet; DE, diet effect; OE, olive oil effect; DExOE, interaction between diet and olive oil; Fas, fatty acid synthase; NASH, non-alcoholic steatohepatitis; NS, not significant; Pparγ, peroxisome proliferator-activated receptor gamma; Scd1, stearoyl- coenzyme A desaturase 1; Srebp1c, sterol regulatory element-binding protein 1c.

^a^ p<0.05 compared to C-fed mice.

^c^ p<0.05 compared to CO-fed mice.

^**˄**^Values are means ± SEM, n = 7–8, except for PPARγ activity: n = 3–4.

### Effect of extra virgin olive oil on markers of TLR4 signaling in liver tissue

To determine if olive oil intake affected markers of toll-like receptor 4 (Tlr4) signaling shown to be critical in the development of NAFLD [40, 41], expression of *Tlr4*, myeloid differentiation factor 88 *(Myd88*) and lipopolysaccharide binding protein (*Lbp*) were assessed in liver tissue of animals. Expressions of *Tlr4* mRNA were significantly higher in livers of BFC- and OFC-fed mice than in C-fed animals while for the comparison with CO-fed mice, only differences between BFC-fed and C-fed mice reach the level of significance as data varied considerably in some groups (see Table 5). Contrasting these findings, neither mRNA expression of *Myd88* or *Lbp* differed between groups.

**Table 5:** Effect of olive oil on parameters of TLR4 signaling in female mice with early NASH.

Parameter	13 weeks^˄^				Two-way ANOVA		
	C	BFC	CO	OFC	DEx OE	OE	DE
***Tlr4* mRNA (% of control)**	100.0 ± 7.0	227.5 ± 29.7^a^^,^^c^	116.4 ± 17.6	190.7 ± 21.2^a^	NS	NS	<0.05
***Myd88* mRNA (% of control)**	100.0 ± 11.1	89.2 ± 9.5	85.8 ± 8.9	98.4 ± 15.7	NS	NS	NS
***Lbp* mRNA (% of control)**	100.0 ± 8.6	112.4 ± 16.3	68.5 ± 13.2	115.7 ± 22.4	NS	NS	NS

C, control diet; BFC, butterfat-, fructose- and cholesterol-rich diet; CO, control diet with extra virgin olive oil; OFC, extra virgin olive oil-, fructose- and cholesterol-rich diet; DE, diet effect; OE, olive oil effect; DExOE, interaction between diet and olive oil; NASH, non-alcoholic steatohepatitis; NS, not significant; Tlr4, toll-like receptor 4; Myd88, myeloid differentiation factor 88; Lbp, lipopolysaccharide binding protein.

^a^ p<0.05 compared to C-fed mice.

^c^ p<0.05 compared to CO-fed mice.

^**˄**^Values are means ± SEM, n = 7–8.

## Discussion

The impact of macronutrients and herein especially of different fats and oils on the development of metabolic diseases including insulin resistance and NAFLD is still not fully understood. Life-style interventions aiming at a weight loss are still the first therapy of choice in the treatment of metabolic diseases. These interventions often focus on a general reduction of caloric intake e.g. through a reduction of fat intake and increas of the intake of complex carbohydrate and/or recommend a change of dietary pattern towards a `Mediterranean diet´ or modification of the latter often associated with a switch of fat source to olive oil [42–44]. Some human and animal studies suggest that olive oil may bear some beneficial effects on the development of insulin resistance and NAFLD even in the absence of other dietary factors associated with the consumption of a `Mediterranean diet´ [45, 46]; however, results of other studies report no or even unfavorable health effects of olive oil [23, 24]. Here, using a pair-feeding model in mice, we assessed, if exchanging butterfat with equivalent amounts of an extra virgin olive oil bears protective effects on the progression of insulin resistance and/or development of NAFLD. Indeed, when compared to butterfat, extra virgin olive oil is markedly richer in oleic acids (18:1: olive oil 76–78% vs. 2.58% in butterfat) but also some polyunsaturated fatty acids (18:2: olive oil 4.5–6% vs. 0.21% in butterfat; 18:3: olive oil 0.5–0.7% vs. 0.06% in butterfat). In the present study, while only slightly differing in absolute body weight gain, mice fed the BFC and those fed the OFC for the last 5 weeks of the experiment were similar regarding markers of insulin resistance and early non-alcoholic steatohepatitis (NASH). Indeed, with the exception of AST activity, none of the parameters assessed related to liver health, e.g. NAS and ALT activity or related to insulin resistance (e.g. insulin tolerance, glucose tolerance, mRNA expression of *Ir* and *Irs2* in muscle or liver tissue) differed between mice fed the BFC or OFC diet. However, almost all of these parameters were significantly higher in BFC and OFC animals when compared to the two control groups. Also, mRNA expressions of proinflammatory cytokines like *Tnfα* and *Il-6* as well as concentrations of 4-HNE protein adducts were also similarly elevated in livers of BFC- and OFC-fed mice. Furthermore, while mRNA expression of *Bad* was lower in livers of both BFC- and OFC-fed mice, cleaved caspase 3 was similar between groups as concentration of the latter varied considerably between groups. The pro-apoptotic protein Bad has been shown to be also critical in the regulation of hepatic energy metabolism [47]. And while others have shown that pro-apoptotic markers like Bad are upregulated in settings of NAFLD [48, 49], it could very well be that in the present study, the lower expression of *Bad* found in livers of OFC- and BFC-fed mice was related to its role regulating gluconeogensis; however, this remains to be determined in future studies. Interestingly, markers of lipogenesis were also similarly altered in livers of BFC- and OFC-fed mice further suggesting that replacing butterfat with similar amounts of extra virgin olive oil only had limited effects on lipogenesis in liver in the present study. Findings for *Srebp1c* mRNA expression as well as PPARγ activity and expression in liver are somewhat contrasting the findings of others reporting that in settings of high-fat diet/ obesity-associated NAFLD, these nuclear factors are found to be increased [50–52]. Difference between the findings of others and those in the present study might have resulted from the lack of abnormal body weight in the present study but maybe also differences in diets fed as well as gender. This also needs to be addressed in future studies. Furthermore, the difference found in AST activity may have resulted from the fact that AST is considered as less specific for liver injury but rather may also be indicative of other tissues damaged e.g. heart, pancreas and muscle [53]. Indeed, ALT activity is considered as a better indicator of liver damage as its activity is greater in liver than in muscle (for overview see [54]).

Taken together, results of the present study suggest, that the simple exchange of the source of fat e.g. from butterfat to extra virgin olive oil when continuously feeding a diet rich in fructose and cholesterol but also fat, may not be sufficient to attenuate the progression of NAFLD and insulin resistance in mice. Rather, our data suggest that the fatty acid composition and polyphenols found in a commercially available extra virgin olive oil may not be sufficient to attenuate NAFLD progression. However, these findings by no means preclude that extra virgin olive oil when being fed concomitantly from the beginning or when being fed in the absence of fructose and/or cholesterol may prevent the development and progression of NAFLD.

Our results are in part contrary to the findings of others assessing the effect of olive oil on the development of NAFLD and/or insulin resistance. For instance, Sales et al. [55] reported that an 8 weeks long intake of an olive oil rich diet (41E% fat) did not resulted in the development of steatosis in mice while that of palm oil did. Furthermore, in the study of Jurado-Ruiz et al. [22] assessing the effect of extra virgin olive oil on the progression of NAFLD induced by feeding mice a lard-based high-fat diet, the exchange of lard with an extra virgin olive oil also partially attenuated the progression of inflammation but not steatosis in mice with pre-existing NAFLD. In line with the findings of the present study, supplementing diet with olive oil in that study was also not associated with changes in IL-6 and TNFα levels in plasma [22]. Also, in the study of Jurado-Ruiz et al. effects on liver were more pronounced in those mice receiving a polyphenol enriched olive oil further suggesting that maybe not the fatty acid pattern but other compounds found in extra virgin olive oil may have beneficial effects on the liver. Contrasting the present study, in that study animals were not pair-fed and especially those in the polyphenol enriched olive oil group lost weight when being fed the olive oil enriched diet suggesting bolstering the hypothesis that the polyphenol content of the olive oil may be critical for the beneficial effects. Indeed, weight loss has been shown to attenuate the development of NAFLD in several human and animal studies [56, 57]. Dos Santos et al. also showed that changing the diet after 8 weeks from a high-fat diet to a high-fat diet supplemented with extra virgin olive oil “slowed” the NAFLD progression with beneficial effects being associated with changes in mitochondrial composition e.g. fatty acids [58]. In the study of Hernández-Rodas et al. the slight beneficial effect of an addition of 50 mg/kg/d extra virgin olive oil to a high-fat diet on liver fat (~-24% compared to high-fat diet-fed mice) were associated with markedly lower *Srebp1c* and *Fas* mRNA expression as well as lower activity of Acc and Fas. Also, in this study, markers of lipid peroxidation as well as expression of proinflammatory cytokines in liver tissue were lower in livers of mice fed the high-fat diet fortified with extra virgin olive oil [59]. Differences between results of this study and the present study might have resulted from the amount of extra virgin olive oil supplemented e.g. 25E% vs. 50 mg/kg/d as well as the experimental setup (concomitant treatment for 12 weeks vs. exchanging fat source after 8 weeks). Indeed, results of Rincón-Cervera et al. also showed that supplementing a high-fat diet with an extra virgin olive oil rich with antioxidants may partially attenuate the hepatic fat accumulation and the development of insulin resistance and that this may be associated with changes in markers of lipid peroxidation. However, results of this study also suggest that not only fatty acid composition but also compounds found in extra virgin olive oil like antioxidants may be important for the effects of the oil on liver health and glucose metabolism in settings of diet-induced NAFLD [60]. In line with this hypothesis, it was shown in rats fed an iron-rich diet that the addition of an antioxidant-rich extra virgin olive oil to the diet (100 mg/d) hindered the development of liver steatosis through mechanisms involving an attenuation of oxidative stress and induction of markers of lipogenesis [61]. Unfortunately, in the present study, no data were available regarding polyphenol or antioxidant content of the extra virgin olive oil.

Results of recent studies suggest that certain extra virgin olive oils may affect intestinal barrier function in settings of inflammatory bowel disease [62]. Impairments of intestinal barrier function, and subsequently, an increased translocation of bacterial endotoxin and activation of TLR4-dependent signaling cascades in the liver have repeatedly been associated with the development of NAFLD (for overview [63]). In line with the findings of our group but also other groups [64–66], in the present study, expression of *Tlr4* in liver tissue of both BFC and OFC was significantly higher than in C-fed animals. Somewhat contrasting the findings for *Tlr4* mRNA expression, expression of *Myd88* and *Lbp* were similar between groups. However, it has been shown that neither LBP nor MyD88 are solely regulated at the level of mRNA expression and also in dependence of different ligands/ compounds (e.g. endotoxin, peptidglycane and proinflammatory cytokines) [67–70]. Difference between the studies of Cariello et al. [62] and our study might have resulted from differences in the composition of the oils used but may also be related to difference in the models (e.g. dextran sodium sulfate vs. diet-induced NAFLD). Taken together, our results suggest that the addition of extra virgin olive oil instead of butterfat had no effects on *Tlr4* expression in liver tissue of mice with diet-induced early signs of NASH. Further studies are needed to determine if specific cultivars of olives and subsequently, different compounds found in the oil or the fatty acid composition found in olive oil are critical in the effects of olive oil on intestinal barrier function.

## Conclusions

Taken together, results of the present study suggest that in settings of a preexisting steatosis with early signs of inflammation and insulin resistance, an exchange of all fat sources from butterfat to an extra virgin olive oil neither attenuates the progression of liver disease nor insulin resistance in mice when the consumption of “unhealthy” nutrients such as fructose and cholesterol is continued. These data support the hypothesis that large amounts of extra virgin olive oil by itself may only have limited therapeutic impact on liver and glucose metabolism in settings of a pre-existing NAFLD and impaired glucose and insulin tolerance. However, if this is a universal effect of extra virgin olive oil or if this is only attributable for the extra virgin olive oil or the amounts used in the present mouse study, remains to be determined.

## Supporting information

S1 Fig Study design. (TIF)

S2 Fig Representative picture of Western blot of cleaved caspase 3 and β-actin. (TIF)

S3 Fig Graphical abstract. (TIF)

S1 Table Composition of diets (Ssniff Spezialdiäten GmbH, Soest, Germany). (PDF)

S2 Table Primer sequences used for real-time RT-PCR. (PDF)

S1 Raw images (PDF)

## References

[1] Ullah R, Rauf N, Nabi G, Ullah H, Shen Y, Zhou YD, et al Role of Nutrition in the Pathogenesis and Prevention of Non-alcoholic Fatty Liver Disease: Recent Updates. Int J Biol Sci. 2019;15(2):265–76. 10.7150/ijbs.30121 30745819 PMC6367556

[2] Sass DA, Chang P, Chopra KB. Nonalcoholic fatty liver disease: a clinical review. Dig Dis Sci. 2005;50(1):171–80. 10.1007/s10620-005-1267-z . 15712657

[3] Younossi ZM, Koenig AB, Abdelatif D, Fazel Y, Henry L, Wymer M. Global epidemiology of nonalcoholic fatty liver disease-Meta-analytic assessment of prevalence, incidence, and outcomes. Hepatology. 2016;64(1):73–84. 10.1002/hep.28431 . 26707365

[4] Younossi ZM, Golabi P, de Avila L, Paik JM, Srishord M, Fukui N, et al The global epidemiology of NAFLD and NASH in patients with type 2 diabetes: A systematic review and meta-analysis. J Hepatol. 2019;71(4):793–801. 10.1016/j.jhep.2019.06.021 . 31279902

[5] Pais R, Barritt ASt, Calmus Y, Scatton O, Runge T, Lebray P, et al NAFLD and liver transplantation: Current burden and expected challenges. J Hepatol. 2016;65(6):1245–57. 10.1016/j.jhep.2016.07.033 27486010 PMC5326676

[6] Younossi ZM, Blissett D, Blissett R, Henry L, Stepanova M, Younossi Y, et al The economic and clinical burden of nonalcoholic fatty liver disease in the United States and Europe. Hepatology. 2016;64(5):1577–86. 10.1002/hep.28785 . 27543837

[7] McManus K, Antinoro L, Sacks F. A randomized controlled trial of a moderate-fat, low-energy diet compared with a low fat, low-energy diet for weight loss in overweight adults. Int J Obes Relat Metab Disord. 2001;25(10):1503–11. 10.1038/sj.ijo.0801796 . 11673773

[8] Tomic D, Kemp WW, Roberts SK. Nonalcoholic fatty liver disease: current concepts, epidemiology and management strategies. Eur J Gastroenterol Hepatol. 2018;30(10):1103–15. 10.1097/MEG.0000000000001235 . 30113367

[9] Riazi K, Raman M, Taylor L, Swain MG, Shaheen AA. Dietary Patterns and Components in Nonalcoholic Fatty Liver Disease (NAFLD): What Key Messages Can Health Care Providers Offer? Nutrients. 2019;11(12). 10.3390/nu11122878 31779112 PMC6950597

[10] Chung GE, Youn J, Kim YS, Lee JE, Yang SY, Lim JH, et al Dietary patterns are associated with the prevalence of nonalcoholic fatty liver disease in Korean adults. Nutrition. 2019;62:32–8. 10.1016/j.nut.2018.11.021 . 30826597

[11] Mirmiran P, Amirhamidi Z, Ejtahed HS, Bahadoran Z, Azizi F. Relationship between Diet and Non-alcoholic Fatty Liver Disease: A Review Article. Iran J Public Health. 2017;46(8):1007–17. 28894701 PMC5575379

[12] Zelber-Sagi S, Ivancovsky-Wajcman D, Fliss Isakov N, Webb M, Orenstein D, Shibolet O, et al High red and processed meat consumption is associated with non-alcoholic fatty liver disease and insulin resistance. J Hepatol. 2018;68(6):1239–46. 10.1016/j.jhep.2018.01.015 . 29571924

[13] Cordain L, Eaton SB, Sebastian A, Mann N, Lindeberg S, Watkins BA, et al Origins and evolution of the Western diet: health implications for the 21st century. Am J Clin Nutr. 2005;81(2):341–54. 10.1093/ajcn.81.2.341 . 15699220

[14] Parker HM, Johnson NA, Burdon CA, Cohn JS, O'Connor HT, George J. Omega-3 supplementation and non-alcoholic fatty liver disease: a systematic review and meta-analysis. J Hepatol. 2012;56(4):944–51. 10.1016/j.jhep.2011.08.018 . 22023985

[15] Yan JH, Guan BJ, Gao HY, Peng XE. Omega-3 polyunsaturated fatty acid supplementation and non-alcoholic fatty liver disease: A meta-analysis of randomized controlled trials. Medicine (Baltimore). 2018;97(37):e12271 10.1097/MD.0000000000012271 30212963 PMC6155966

[16] Khalatbari-Soltani S, Imamura F, Brage S, De Lucia Rolfe E, Griffin SJ, Wareham NJ, et al The association between adherence to the Mediterranean diet and hepatic steatosis: cross-sectional analysis of two independent studies, the UK Fenland Study and the Swiss CoLaus Study. BMC Med. 2019;17(1):19 10.1186/s12916-019-1251-7 30674308 PMC6345041

[17] Abenavoli L, Milanovic M, Milic N, Luzza F, Giuffre AM. Olive oil antioxidants and non-alcoholic fatty liver disease. Expert Rev Gastroenterol Hepatol. 2019;13(8):739–49. 10.1080/17474124.2019.1634544 . 31215262

[18] Widmer RJ, Flammer AJ, Lerman LO, Lerman A. The Mediterranean diet, its components, and cardiovascular disease. Am J Med. 2015;128(3):229–38. Epub 2014/12/03. 10.1016/j.amjmed.2014.10.014 25447615 PMC4339461

[19] Foscolou A, Critselis E, Panagiotakos D. Olive oil consumption and human health: A narrative review. Maturitas. 2018;118:60–6. 10.1016/j.maturitas.2018.10.013 . 30415757

[20] Assy N, Nassar F, Nasser G, Grosovski M. Olive oil consumption and non-alcoholic fatty liver disease. World journal of gastroenterology. 2009;15(15):1809–15. 10.3748/wjg.15.1809 19370776 PMC2670406

[21] Valenzuela R, Videla LA. Impact of the Co-Administration of N-3 Fatty Acids and Olive Oil Components in Preclinical Nonalcoholic Fatty Liver Disease Models: A Mechanistic View. Nutrients. 2020;12(2). Epub 2020/02/23. 10.3390/nu12020499 32075238 PMC7071322

[22] Jurado-Ruiz E, Varela LM, Luque A, Berna G, Cahuana G, Martinez-Force E, et al An extra virgin olive oil rich diet intervention ameliorates the nonalcoholic steatohepatitis induced by a high-fat "Western-type" diet in mice. Mol Nutr Food Res. 2017;61(3). 10.1002/mnfr.201600549 . 27749006

[23] Li X, Cui K, Fang W, Chen Q, Xu D, Mai K, et al High level of dietary olive oil decreased growth, increased liver lipid deposition and induced inflammation by activating the p38 MAPK and JNK pathways in large yellow croaker (Larimichthys crocea). Fish Shellfish Immunol. 2019;94:157–65. Epub 2019/08/30. 10.1016/j.fsi.2019.08.062 . 31465874

[24] Harari A, Leikin Frenkel A, Barshack I, Sagee A, Cohen H, Kamari Y, et al Addition of fish oil to atherogenic high fat diet inhibited atherogenesis while olive oil did not, in LDL receptor KO mice. Nutr Metab Cardiovasc Dis. 2020;30(4):709–16. Epub 2020/02/03. 10.1016/j.numecd.2019.12.007 . 32007335

[25] Sellmann C, Baumann A, Brandt A, Jin CJ, Nier A, Bergheim I. Oral Supplementation of Glutamine Attenuates the Progression of Nonalcoholic Steatohepatitis in C57BL/6J Mice. J Nutr. 2017;147(11):2041–9. Epub 2017/09/22. 10.3945/jn.117.253815 . 28931589

[26] Spruss A, Henkel J, Kanuri G, Blank D, Puschel GP, Bischoff SC, et al Female mice are more susceptible to nonalcoholic fatty liver disease: sex-specific regulation of the hepatic AMP-activated protein kinase-plasminogen activator inhibitor 1 cascade, but not the hepatic endotoxin response. Mol Med. 2012;18:1346–55. Epub 2012/09/07. 10.2119/molmed.2012.00223 22952059 PMC3521787

[27] Marin V, Rosso N, Dal Ben M, Raseni A, Boschelle M, Degrassi C, et al An Animal Model for the Juvenile Non-Alcoholic Fatty Liver Disease and Non-Alcoholic Steatohepatitis. PloS one. 2016;11(7):e0158817 Epub 2016/07/09. 10.1371/journal.pone.0158817 27391242 PMC4938400

[28] Prendergast BJ, Onishi KG, Zucker I. Female mice liberated for inclusion in neuroscience and biomedical research. Neurosci Biobehav Rev. 2014;40:1–5. 10.1016/j.neubiorev.2014.01.001 . 24456941

[29] Baumann A, Jin CJ, Brandt A, Sellmann C, Nier A, Burkard M, et al Oral Supplementation of Sodium Butyrate Attenuates the Progression of Non-Alcoholic Steatohepatitis. Nutrients. 2020;12(4). Epub 2020/04/03. 10.3390/nu12040951 32235497 PMC7231312

[30] Jin CJ, Sellmann C, Engstler AJ, Ziegenhardt D, Bergheim I. Supplementation of sodium butyrate protects mice from the development of non-alcoholic steatohepatitis (NASH). Br J Nutr. 2015;114(11):1745–55. 10.1017/S0007114515003621 . 26450277

[31] Kleiner DE, Brunt EM, Van Natta M, Behling C, Contos MJ, Cummings OW, et al Design and validation of a histological scoring system for nonalcoholic fatty liver disease. Hepatology. 2005;41(6):1313–21. 10.1002/hep.20701 . 15915461

[32] Brandt A, Jin CJ, Nolte K, Sellmann C, Engstler AJ, Bergheim I. Short-Term Intake of a Fructose-, Fat- and Cholesterol-Rich Diet Causes Hepatic Steatosis in Mice: Effect of Antibiotic Treatment. Nutrients. 2017;9(9). 10.3390/nu9091013 28906444 PMC5622773

[33] Sellmann C, Priebs J, Landmann M, Degen C, Engstler AJ, Jin CJ, et al Diets rich in fructose, fat or fructose and fat alter intestinal barrier function and lead to the development of nonalcoholic fatty liver disease over time. J Nutr Biochem. 2015;26(11):1183–92. 10.1016/j.jnutbio.2015.05.011 . 26168700

[34] Spruss A, Kanuri G, Stahl C, Bischoff SC, Bergheim I. Metformin protects against the development of fructose-induced steatosis in mice: role of the intestinal barrier function. Lab Invest. 2012;92(7):1020–32. 10.1038/labinvest.2012.75 . 22525431

[35] Wagnerberger S, Spruss A, Kanuri G, Volynets V, Stahl C, Bischoff SC, et al Toll-like receptors 1–9 are elevated in livers with fructose-induced hepatic steatosis. Br J Nutr. 2012;107(12):1727–38. 10.1017/S0007114511004983 . 22018861

[36] Baulies A, Ribas V, Nunez S, Torres S, Alarcon-Vila C, Martinez L, et al Lysosomal Cholesterol Accumulation Sensitizes To Acetaminophen Hepatotoxicity by Impairing Mitophagy. Sci Rep. 2015;5:18017 Epub 2015/12/15. 10.1038/srep18017 26657973 PMC4676017

[37] Brandt A, Nier A, Jin CJ, Baumann A, Jung F, Ribas V, et al Consumption of decaffeinated coffee protects against the development of early non-alcoholic steatohepatitis: Role of intestinal barrier function. Redox Biol. 2019;21:101092 10.1016/j.redox.2018.101092 30605883 PMC6313826

[38] Sellmann C, Jin CJ, Engstler AJ, De Bandt JP, Bergheim I. Oral citrulline supplementation protects female mice from the development of non-alcoholic fatty liver disease (NAFLD). Eur J Nutr. 2017;56(8):2519–27. 10.1007/s00394-016-1287-9 . 27496089

[39] Chen L, Willis SN, Wei A, Smith BJ, Fletcher JI, Hinds MG, et al Differential targeting of prosurvival Bcl-2 proteins by their BH3-only ligands allows complementary apoptotic function. Mol Cell. 2005;17(3):393–403. Epub 2005/02/08. 10.1016/j.molcel.2004.12.030 . 15694340

[40] Jin CJ, Engstler AJ, Ziegenhardt D, Bischoff SC, Trautwein C, Bergheim I. Loss of lipopolysaccharide-binding protein attenuates the development of diet-induced non-alcoholic fatty liver disease in mice. Journal of gastroenterology and hepatology. 2017;32(3):708–15. Epub 2016/07/13. 10.1111/jgh.13488 . 27404046

[41] Spruss A, Kanuri G, Wagnerberger S, Haub S, Bischoff SC, Bergheim I. Toll-like receptor 4 is involved in the development of fructose-induced hepatic steatosis in mice. Hepatology. 2009;50(4):1094–104. Epub 2009/07/29. 10.1002/hep.23122 . 19637282

[42] Zelber-Sagi S, Salomone F, Mlynarsky L. The Mediterranean dietary pattern as the diet of choice for non-alcoholic fatty liver disease: Evidence and plausible mechanisms. Liver Int. 2017;37(7):936–49. 10.1111/liv.13435 . 28371239

[43] European Association for the Study of the L, European Association for the Study of D, European Association for the Study of O. EASL-EASD-EASO Clinical Practice Guidelines for the management of non-alcoholic fatty liver disease. J Hepatol. 2016;64(6):1388–402. 10.1016/j.jhep.2015.11.004 . 27062661

[44] Esposito K, Maiorino MI, Petrizzo M, Bellastella G, Giugliano D. The effects of a Mediterranean diet on the need for diabetes drugs and remission of newly diagnosed type 2 diabetes: follow-up of a randomized trial. Diabetes Care. 2014;37(7):1824–30. Epub 2014/04/12. 10.2337/dc13-2899 . 24722497

[45] Rezaei S, Akhlaghi M, Sasani MR, Barati Boldaji R. Olive oil lessened fatty liver severity independent of cardiometabolic correction in patients with non-alcoholic fatty liver disease: A randomized clinical trial. Nutrition. 2019;57:154–61. 10.1016/j.nut.2018.02.021 . 30170304

[46] Jurado-Ruiz E, Alvarez-Amor L, Varela LM, Berna G, Parra-Camacho MS, Oliveras-Lopez MJ, et al Extra virgin olive oil diet intervention improves insulin resistance and islet performance in diet-induced diabetes in mice. Sci Rep. 2019;9(1):11311 Epub 2019/08/07. 10.1038/s41598-019-47904-z PubMed Central PMCID: PMC6683141. 31383924 PMC6683141

[47] Gimenez-Cassina A, Garcia-Haro L, Choi CS, Osundiji MA, Lane EA, Huang H, et al Regulation of hepatic energy metabolism and gluconeogenesis by BAD. Cell Metab. 2014;19(2):272–84. Epub 2014/02/11. 10.1016/j.cmet.2013.12.001 PubMed Central PMCID: PMC3971904. 24506868 PMC3971904

[48] Amali AA, Rekha RD, Lin CJ, Wang WL, Gong HY, Her GM, et al Thioacetamide induced liver damage in zebrafish embryo as a disease model for steatohepatitis. J Biomed Sci. 2006;13(2):225–32. Epub 2006/02/04. 10.1007/s11373-005-9055-5 16456712

[49] Li CP, Li JH, He SY, Li P, Zhong XL. Roles of Fas/Fasl, Bcl-2/Bax, and Caspase-8 in rat nonalcoholic fatty liver disease pathogenesis. Genet Mol Res. 2014;13(2):3991–9. Epub 2014/06/19. 10.4238/2014.May.23.10 24938610

[50] Matsusue K, Haluzik M, Lambert G, Yim SH, Gavrilova O, Ward JM, et al Liver-specific disruption of PPARgamma in leptin-deficient mice improves fatty liver but aggravates diabetic phenotypes. J Clin Invest. 2003;111(5):737–47. Epub 2003/03/06. 10.1172/JCI17223 PubMed Central PMCID: PMC151902. 12618528 PMC151902

[51] Wang F, Mullican SE, DiSpirito JR, Peed LC, Lazar MA. Lipoatrophy and severe metabolic disturbance in mice with fat-specific deletion of PPARgamma. Proc Natl Acad Sci U S A. 2013;110(46):18656–61. Epub 2013/10/30. 10.1073/pnas.1314863110 24167256 PMC3831974

[52] Yamazaki T, Shiraishi S, Kishimoto K, Miura S, Ezaki O. An increase in liver PPARgamma2 is an initial event to induce fatty liver in response to a diet high in butter: PPARgamma2 knockdown improves fatty liver induced by high-saturated fat. J Nutr Biochem. 2011;22(6):543–53. Epub 2010/08/31. 10.1016/j.jnutbio.2010.04.009 . 20801631

[53] Giannini EG, Testa R, Savarino V. Liver enzyme alteration: a guide for clinicians. CMAJ. 2005;172(3):367–79. Epub 2005/02/03. 10.1503/cmaj.1040752 15684121 PMC545762

[54] McGill MR. The past and present of serum aminotransferases and the future of liver injury biomarkers. EXCLI J. 2016;15:817–28. Epub 2016/01/01. 10.17179/excli2016-800 28337112 PMC5318690

[55] Sales RC, Medeiros PC, Spreafico F, de Velasco PC, Goncalves FKA, Martin-Hernandez R, et al Olive Oil, Palm Oil, and Hybrid Palm Oil Distinctly Modulate Liver Transcriptome and Induce NAFLD in Mice Fed a High-Fat Diet. Int J Mol Sci. 2018;20(1). 10.3390/ijms20010008 30577497 PMC6337378

[56] Vilar-Gomez E, Martinez-Perez Y, Calzadilla-Bertot L, Torres-Gonzalez A, Gra-Oramas B, Gonzalez-Fabian L, et al Weight Loss Through Lifestyle Modification Significantly Reduces Features of Nonalcoholic Steatohepatitis. Gastroenterology. 2015;149(2):367–78 e5; quiz e14-5. 10.1053/j.gastro.2015.04.005 . 25865049

[57] Mendes IKS, Matsuura C, Aguila MB, Daleprane JB, Martins MA, Mury WV, et al Weight loss enhances hepatic antioxidant status in a NAFLD model induced by high-fat diet. Appl Physiol Nutr Metab. 2018;43(1):23–9. Epub 2017/08/24. 10.1139/apnm-2017-0317 . 28834687

[58] dos Santos GA, Ferreira MS, de Oliveira DN, de Oliveira V, Siqueira-Santos ES, Cintra DE, et al Identification of compounds from high-fat and extra virgin olive oil-supplemented diets in whole mouse liver extracts and isolated mitochondria using mass spectrometry. J Mass Spectrom. 2015;50(7):951–8. 10.1002/jms.3609 . 26349651

[59] Hernandez-Rodas MC, Valenzuela R, Echeverria F, Rincon-Cervera MA, Espinosa A, Illesca P, et al Supplementation with Docosahexaenoic Acid and Extra Virgin Olive Oil Prevents Liver Steatosis Induced by a High-Fat Diet in Mice through PPAR-alpha and Nrf2 Upregulation with Concomitant SREBP-1c and NF-kB Downregulation. Mol Nutr Food Res. 2017;61(12). 10.1002/mnfr.201700479 . 28940752

[60] Rincon-Cervera MA, Valenzuela R, Hernandez-Rodas MC, Marambio M, Espinosa A, Mayer S, et al Supplementation with antioxidant-rich extra virgin olive oil prevents hepatic oxidative stress and reduction of desaturation capacity in mice fed a high-fat diet: Effects on fatty acid composition in liver and extrahepatic tissues. Nutrition. 2016;32(11–12):1254–67. 10.1016/j.nut.2016.04.006 . 27346714

[61] Barrera C, Valenzuela R, Rincon MA, Espinosa A, Lopez-Arana S, Gonzalez-Manan D, et al Iron-induced derangement in hepatic Delta-5 and Delta-6 desaturation capacity and fatty acid profile leading to steatosis: Impact on extrahepatic tissues and prevention by antioxidant-rich extra virgin olive oil. Prostaglandins Leukot Essent Fatty Acids. 2020;153:102058 Epub 2020/02/03. 10.1016/j.plefa.2020.102058 . 32007744

[62] Cariello M, Contursi A, Gadaleta RM, Piccinin E, De Santis S, Piglionica M, et al Extra-Virgin Olive Oil from Apulian Cultivars and Intestinal Inflammation. Nutrients. 2020;12(4). Epub 2020/04/17. 10.3390/nu12041084 32295122 PMC7230776

[63] Bessone F, Razori MV, Roma MG. Molecular pathways of nonalcoholic fatty liver disease development and progression. Cell Mol Life Sci. 2019;76(1):99–128. Epub 2018/10/22. 10.1007/s00018-018-2947-0 . 30343320 PMC11105781

[64] Sellmann C, Degen C, Jin CJ, Nier A, Engstler AJ, Hasan Alkhatib D, et al Oral arginine supplementation protects female mice from the onset of non-alcoholic steatohepatitis. Amino Acids. 2017;49(7):1215–25. Epub 2017/04/24. 10.1007/s00726-017-2423-4 28434046 PMC5487836

[65] Park S, Choi Y, Um SJ, Yoon SK, Park T. Oleuropein attenuates hepatic steatosis induced by high-fat diet in mice. J Hepatol. 2011;54(5):984–93. Epub 2010/12/15. 10.1016/j.jhep.2010.08.019 . 21145829

[66] Zhou D, Pan Q, Xin FZ, Zhang RN, He CX, Chen GY, et al Sodium butyrate attenuates high-fat diet-induced steatohepatitis in mice by improving gut microbiota and gastrointestinal barrier. World journal of gastroenterology. 2017;23(1):60–75. Epub 2017/01/21. 10.3748/wjg.v23.i1.60 28104981 PMC5221287

[67] Gurung P, Fan G, Lukens JR, Vogel P, Tonks NK, Kanneganti TD. Tyrosine Kinase SYK Licenses MyD88 Adaptor Protein to Instigate IL-1alpha-Mediated Inflammatory Disease. Immunity. 2017;46(4):635–48. Epub 2017/04/16. 10.1016/j.immuni.2017.03.014 28410990 PMC5501252

[68] Kissner TL, Ruthel G, Alam S, Ulrich RG, Fernandez S, Saikh KU. Activation of MyD88 signaling upon staphylococcal enterotoxin binding to MHC class II molecules. PloS one. 2011;6(1):e15985 Epub 2011/02/02. 10.1371/journal.pone.0015985 21283748 PMC3024394

[69] Ciregia F, Baiwir D, Cobraiville G, Dewael T, Mazzucchelli G, Badot V, et al Glycosylation deficiency of lipopolysaccharide-binding protein and corticosteroid-binding globulin associated with activity and response to treatment for rheumatoid arthritis. J Transl Med. 2020;18(1):8 Epub 2020/01/08. 10.1186/s12967-019-02188-9 31907043 PMC6945416

[70] Kirschning C, Unbehaun A, Lamping N, Pfeil D, Herrmann F, Schumann RR. Control of transcriptional activation of the lipopolysaccharide binding protein (LBP) gene by proinflammatory cytokines. Cytokines Cell Mol Ther. 1997;3(1):59–62. Epub 1997/03/01. . 9287245

