# Supplementary figures and images for: Exchanging dietary fat source with extra virgin olive oil does not prevent progression of diet-induced non-alcoholic fatty liver disease and insulin resistance
Source: PLoS One. 2020 Sep 3;15(9):e0237946. doi: 10.1371/journal.pone.0237946 (PMC7470337; doi:10.1371/journal.pone.0237946)

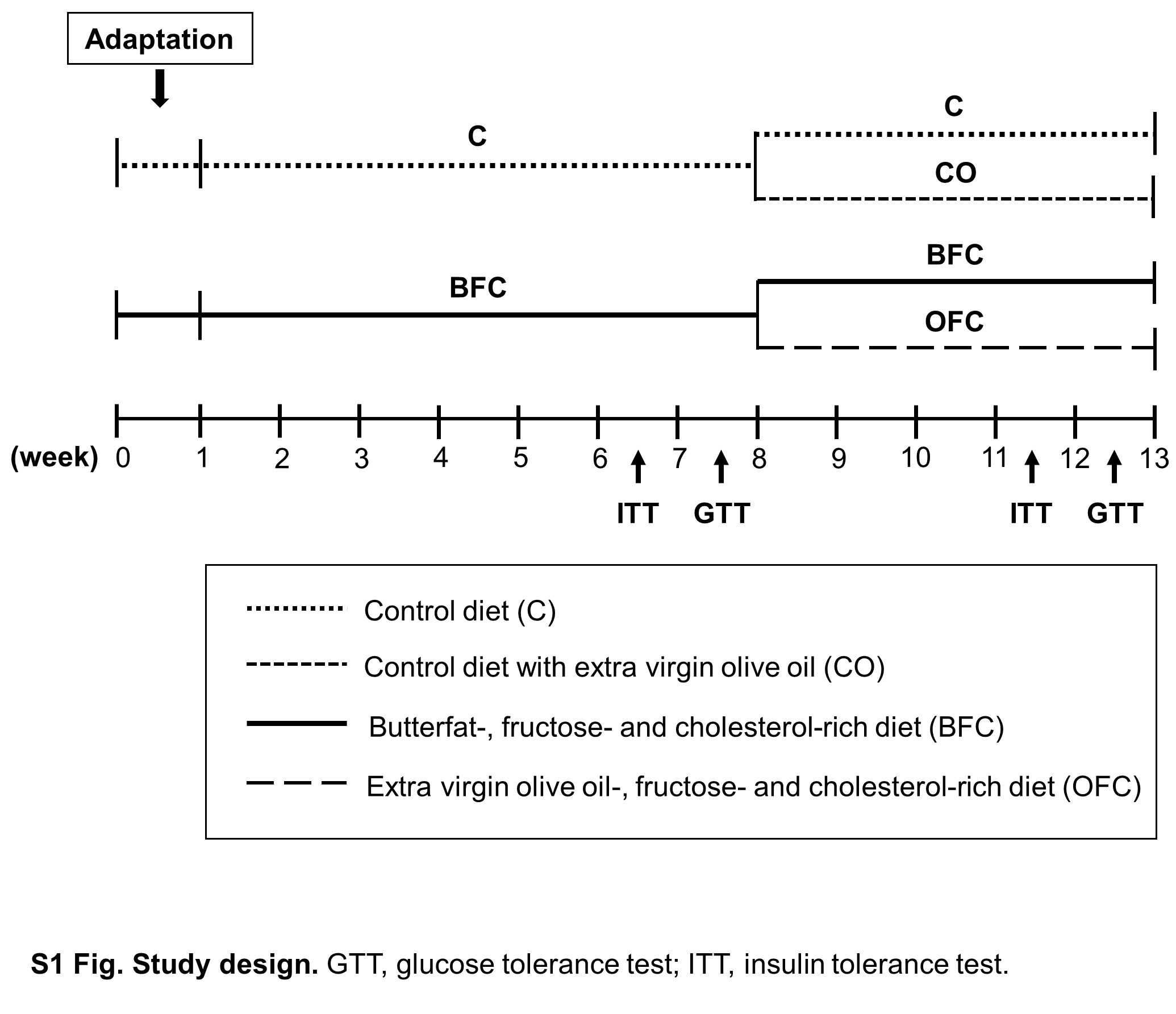

Supplement: S1 Fig — (TIF) [file pone.0237946.s001.tif]

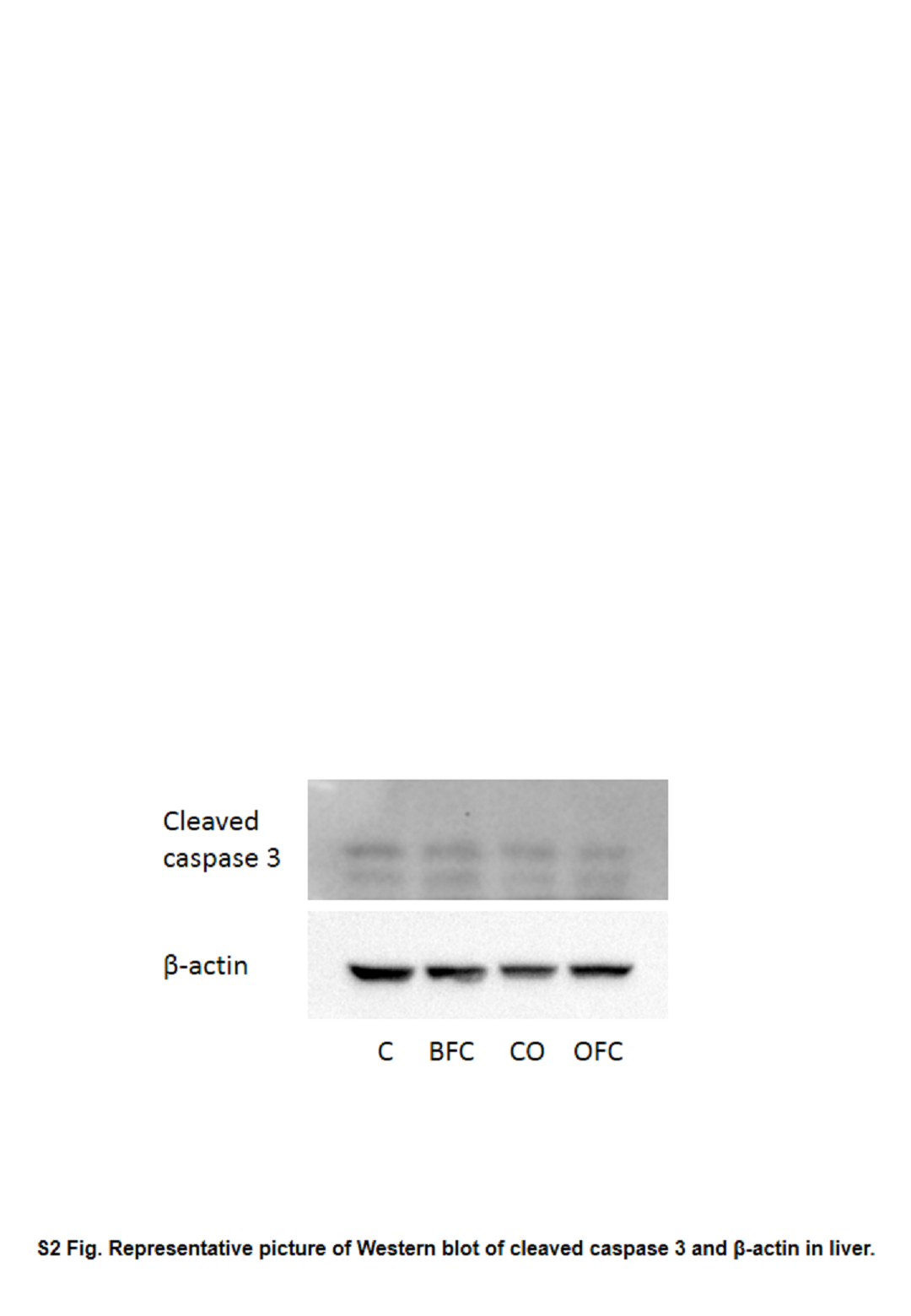

Supplement: S2 Fig — (TIF) [file pone.0237946.s002.tif]

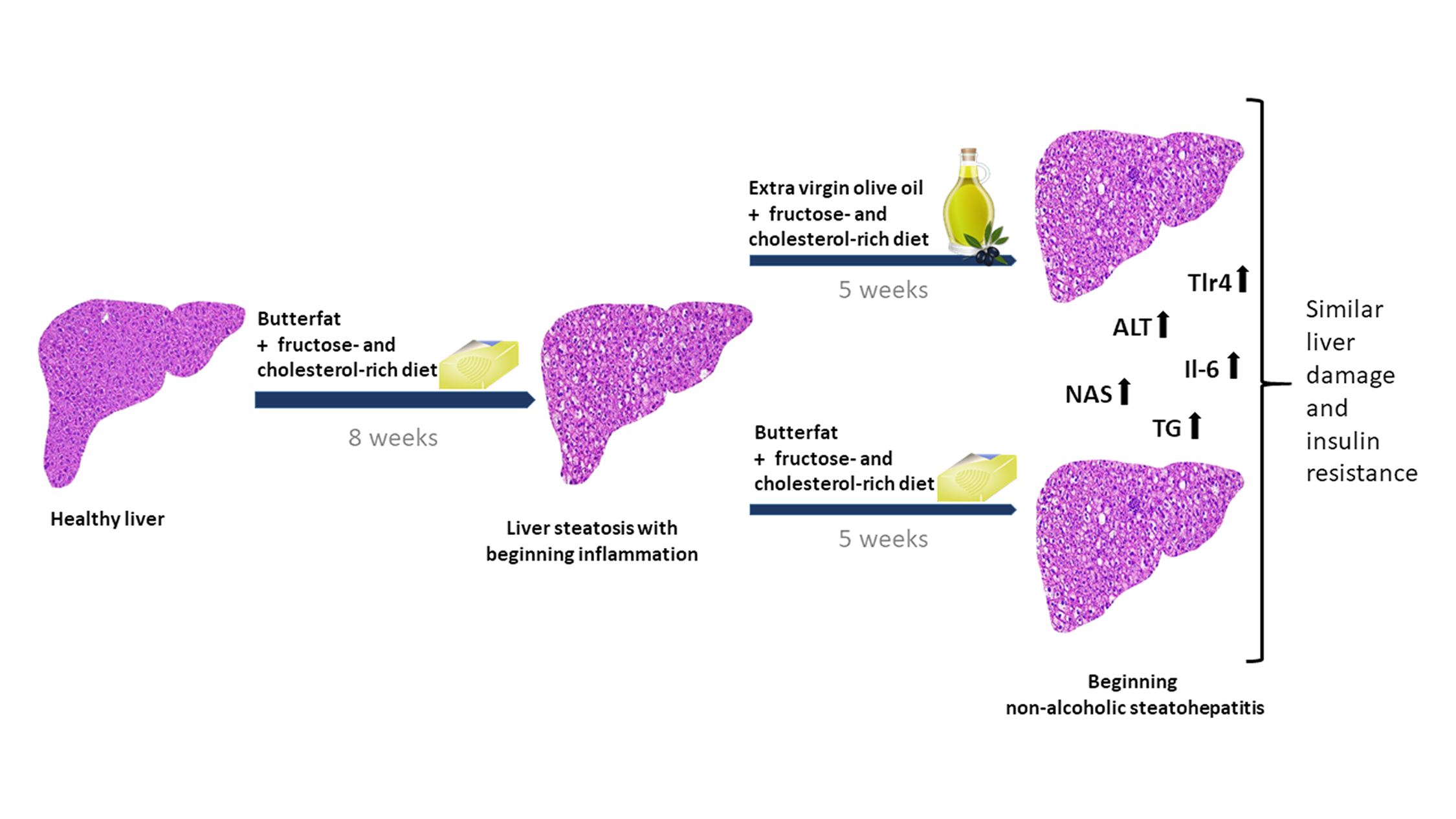

Supplement: S3 Fig — (TIF) [file pone.0237946.s003.tif]
