# Supplementary material for: Exchanging dietary fat source with extra virgin olive oil does not prevent progression of diet-induced non-alcoholic fatty liver disease and insulin resistance
Source: PLoS One. 2020 Sep 3;15(9):e0237946. doi: 10.1371/journal.pone.0237946 (PMC7470337; doi:10.1371/journal.pone.0237946)
Supplement: S1 Table — (PDF) [file pone.0237946.s004.pdf]

S1 Table. Composition of diets (Ssniff Spezialdiäten GmbH, Soest, Germany).

Ingredients	C	CO	BFC	OFC
**Energy (MJ/kg)**	15.7	15.7	17.8	17.8
**Fat (kcal %)**	12	12	25	25
**Protein (kcal %)**	19	19	15	15
**Carbohydrate (kcal %)**	69	69	60	60
**Fructose (% carbohydrate wt/wt)**			50	50
**Cholesterol (% wt/wt)**			0.155	0.155
Fat source (week 1-8)				
**Soybean oil (kcal %)**	12			
**Butterfat (kcal %)**			25	
Fat source (week 9-13)				
**Soybean oil (kcal %)**	12			
**Butterfat (kcal %)**			25	
**Olive oil (kcal %)**		12		25
