# Supplementary material for: Exchanging dietary fat source with extra virgin olive oil does not prevent progression of diet-induced non-alcoholic fatty liver disease and insulin resistance
Source: PLoS One. 2020 Sep 3;15(9):e0237946. doi: 10.1371/journal.pone.0237946 (PMC7470337; doi:10.1371/journal.pone.0237946)
Supplement: S2 Table — (PDF) [file pone.0237946.s005.pdf]

S2 Table. Primer sequences used for real-time RT-PCR.

	Forward (5´- 3´)	Reverse (5´- 3´)
18S	gtaacccgttgaaccccatt	ccatccaatcggtagtagcg
Acc	cttcctcctgatcagcaactct	cgtgagttttcccaaaataagc
Bad	agaccagcagcccagagtatgttc	tgcgcctccatgatgactgttg
Fas	tctgggccaacctcattggt	gaagctgggggtccattgtg
Il-6	ccacgccttccctacttca	tgcaagtgcatcatcgttgttc
Ir	catcccgaaagcgaagatcc	gagtcctgattgcatgcctgcag
Irs2	gaagcggctaagtctcatgg	gacggtggtggtagaggaaa
Lbp	cttggcgtggtcactaatgt	ctcacttgtgccttgtctgg
Myd88	caaaagtggggtgcctttgc	aaatccacagtgcccccaga
Pparγ	aacgtgaagcccatcgagga	ctgcacgtgctctgtgacga
Scd1	ccgataaaagggggctgagg	tgctgagatcgagcgtggac
Srebp1c	accggctactgctggactgc	agagcaagagggtgccatcg
Tlr4	agccattgctgccaacatca	gctgcctcagcaggacttc
Tnfα	cagccaaccaggcaggttct	cctgccacaagcaggaatga

Acc, acetyl- coenzyme A carboxylase; *Bad*, Bcl2-associated agonist of cell death; *Fas*, fatty acid synthase; *Il-6,* interleukin 6; *Ir*, insulin receptor; *Irs2*, insulin receptor substrate 2; *Scd1*, stearoyl- coenzyme A desaturase 1; *Srebp1c*, sterol regulatory element-binding protein 1c; *Lbp,* lipopolysaccharide binding protein; *Myd88,* myeloid differentiation factor 88; *Pparγ*, peroxisome proliferator-activated receptor gamma; *Tlr4*, toll-like receptor 4; *Tnfα*, tumor necrosis factor alpha. Expressions were normalized to *18S* mRNA expression.
