# Supplementary material for: Exchanging dietary fat source with extra virgin olive oil does not prevent progression of diet-induced non-alcoholic fatty liver disease and insulin resistance
Source: PLoS One. 2020 Sep 3;15(9):e0237946. doi: 10.1371/journal.pone.0237946 (PMC7470337; doi:10.1371/journal.pone.0237946)
Supplement: S1 Raw images — (PDF) [file pone.0237946.s006.pdf]

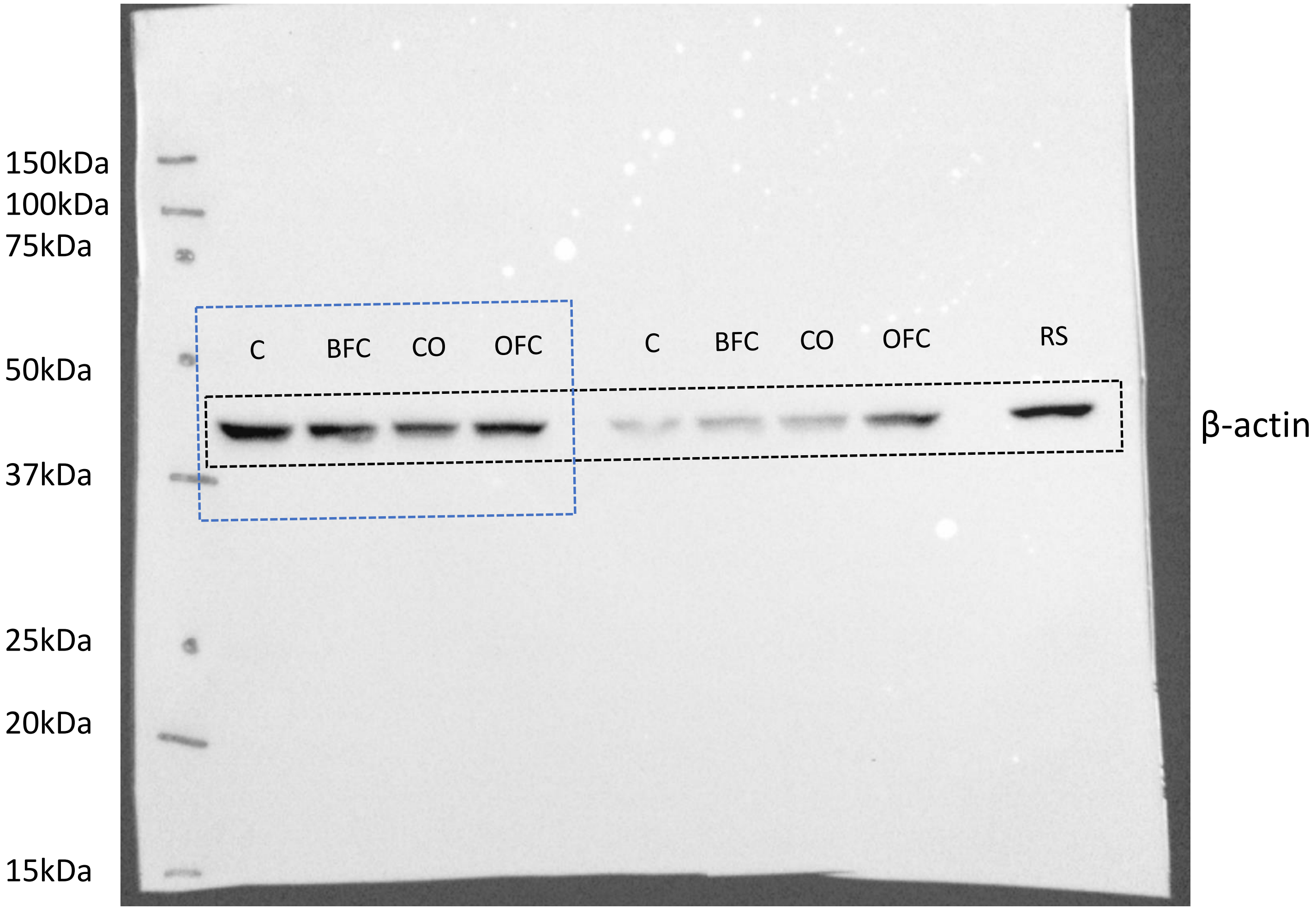

C, control; CO, control diet with olive oil; BFC, butterfat-, fructose- and cholesterol-rich diet; OFC, olive oil-, fructose- and cholesterol-rich diet.
Bands that were highlighted with a blue frame are used for representative picture of Western blot of cleaved caspase 3 and β-actin in liver as shown in S2 Fig.

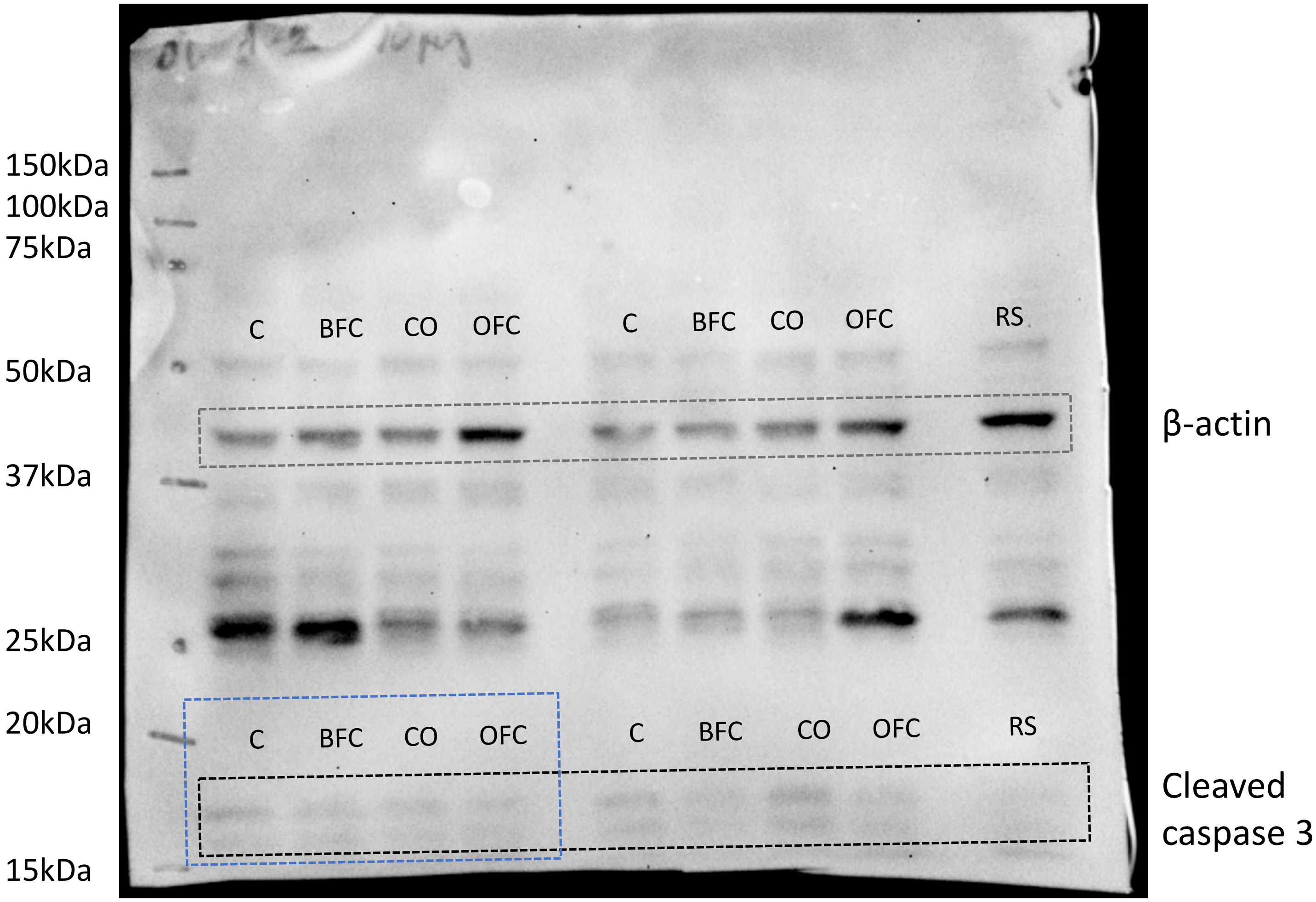

C, control; CO, control diet with olive oil; BFC, butterfat-, fructose- and cholesterol-rich diet; OFC, olive oil-, fructose- and cholesterol-rich diet.
Bands that were highlighted with a blue frame are used for representative picture of Western blot of cleaved caspase 3 and β-actin in liver as shown in S2 Fig.
